# Disability, mental health, stigma and discrimination and neglected tropical diseases

**DOI:** 10.1093/trstmh/traa160

**Published:** 2020-12-12

**Authors:** Hannah Kuper

**Affiliations:** International Centre for Evidence in Disability, London School of Hygiene & Tropical Medicine, Keppel Street, London WC1E 7HT, London, UK

## Abstract

The neglected tropical disease (NTD) agenda should include a focus on disability when ‘planning for the next decade of progress’. Millions of people are currently living with the disabling consequences of NTDs and mental health conditions are frequent among people living with NTDs. Stigma around NTDs is also common. However, these aspects of NTDs are often ignored by programmes that focus on infectious disease control. NTD programmes must broaden in scope to include provision of rehabilitation and linkages to mental health support and tackling stigma through demystifying NTDs. These efforts will promote the inclusion and well-being of people living with NTDs.

The last decade has seen an extraordinary increase in the attention on neglected tropical diseases (NTD). This focus is welcome and needed, given the large burden of NTDs and their concentration among the poorest in the world. It is also unusual in one respect; in contrast to other high-profile conditions like malaria, human immunodeficiency virus and tuberculosis, NTDs generally do not directly kill people. NTDs have therefore attracted attention because they lead to a range of morbidities, and ultimately disability.^1^

Disability arises when someone with a health condition (e.g. *Chlamydia trachomatis* infection) experiences an impairment (e.g. visual loss related to corneal opacity) that causes difficulties in performing activities (e.g. walking independently), potentially resulting in participation restrictions (e.g. inability to work).^2,3^ The pathway from a health condition to exclusion is not inevitable or the same for everyone. It is buffered by personal factors, such as wealth, social support and education, and environmental factors, including the existence of legislation protecting rights or the availability of assistive devices.

If we consider disability in this way, then it is clear that most of the 17 main NTDs can cause impairments, and consequently disability.^2,3^ Trachoma and onchocerciasis can cause blindness; cutaneous/mucocutaneous leishmaniasis, leprosy, chikungunya, yaws, lymphatic filariasis, Buruli ulcer, Chagas disease and African trypanosomiasis can lead to physical impairments; and soil-transmitted helminths and schistosomiasis are linked to delayed physical and mental development and developmental disabilities. Evidence shows that NTDs, by causing impairments, can lead to exclusion from education and learning, reduced employment and economic productivity and poor well-being—in other words, disability.^2,3^

An overlapping issue is that NTDs are also linked to mental health conditions, particularly depression, which has been highlighted in a new World Health Organization (WHO) report ‘Mental health of people with neglected tropical diseases’.^4–6^ The link may be direct and causative, as several NTDs have neurological sequalae.^7^ NTDs may also have an indirect impact on mental health.^4^ Several NTDs, such as leishmaniasis, leprosy and lymphatic filariasis, cause facial and bodily changes, or ‘disfigurements’, that lead to pain and distress.^5^ Exclusion due to physical and sensory impairments may also worsen mental health.^4^ Perhaps most importantly, ‘stigma’ causes both exclusion and poor mental health.^5,6^

Stigma is often an overriding concern of people with NTDs,^5,6^ due to the infectious nature of NTDs and consequent fear of contagion and the obvious physical signs that arise with certain conditions and are often considered unappealing. Stigma sounds simple but actually mixes a range of distinct concepts.^8,9^ The most obvious component is the negative attitudes, often due to fear and ignorance, that people in the community have about someone with an NTD.^5,9^ This can lead to teasing, avoidance, exclusion and even violence. Stigma can also be demonstrated by family members towards the person affected, causing within-household deprivation and exclusion. Self-stigma arises when the affected person is ashamed and holds negative attitudes about his/her value, which may reduce the propensity to reveal symptoms, seek care, demand rights or try to be included in the household or community. Stigma may extend from the person affected to the whole household. Stigma, in whatever form, can therefore perpetuate the exclusion, disability and poor mental health of people with NTDs.^6^

The global initiatives to eliminate NTDs are therefore important, as they will help to avoid mental ill health, stigma, impairments and the consequent exclusions that may occur. The corollary is that NTD programmes will preserve the health and well-being of millions. As one example, the Global Programme to Eliminate Lymphatic Filariasis is estimated to have prevented or cured 96.7 million cases between 2000–2012, people who otherwise could have faced disability and discrimination.^10^ However, fulfilling this mission of NTD elimination will not occur overnight, and there are already millions of people living with disabilities related to NTDs around the world. A key priority in ‘Neglected Tropical Diseases: Planning for the Next Decade of Progress’ should therefore be for the NTD agenda to widen its scope to address disability experienced by people living with NTDs. These efforts can include the provision of mental health support, and the new WHO report offers practical guidance for how this can be achieved.^6^ Other aspects such as provision of rehabilitation (e.g. physiotherapy) and assistive devices should also become part of NTD programmes.^2,3^ More broadly, programmes can include a focus on promoting livelihood and education inclusion and social participation or establishing self-help groups to foster empowerment.^2,3^ Renewed efforts are also needed to address stigma. There are a number of tried and tested strategies for reducing stigma, which mostly focus on demystifying NTDs through contact events, education and media campaigns.^8^ There are examples of these initiatives with respect to leprosy programmes, but for the most part these components are currently lacking in most NTD programmes.^2,3^

There are at least three reasons for these gaps. First, the focus of NTD programmes has been on elimination and eradication, so rehabilitation, whether defined in a narrow medical sense or more holistically and broadly, is not part of the long-term strategy. Second, the lack of data and tools to estimate the link between NTDs and disability^1^ or the effectiveness of interventions to alleviate disability hampers efforts to plan and advocate for these initiatives. Third, NTD programmes are often oriented towards a medical model of disease, identifying and treating clinical symptoms, and so do not focus on the wider repercussions of disease in peoples’ lives. The Next Decade of Progress should work to change this trajectory to recognise the importance of disability and make plans to expand the focus on rehabilitation as part of NTD programmes. NTD programmes should also provide, or strengthen links with, mental health services to enhance the well-being of people living with NTDs, as advocated in the WHO report.^6^

A further consideration is that NTD programmes should ensure that they are disability inclusive, as there are one billion people with disabilities globally. Moreover, disability is particularly prevalent in poor countries and poor communities, which is also where NTDs are concentrated. Yet people with disabilities often face a range of barriers in accessing NTD programmes (e.g. physical inaccessibility, lack of money and stigma), which is a violation of their rights as set out in the United Nations Convention on the Rights of Persons with Disabilities. Making programmes disability inclusive is not necessarily difficult, but requires planning, funding and collaboration with organizations of persons with disabilities and disability-focussed non-governmental organizations.

The extraordinary success of the global NTD programme is to be applauded. In the next decade, NTD programmes should expand their focus to include disability, mental health and stigma, so that further benefits can be reaped by people living with NTDs and disabled people at risk of NTDs.

## Data Availability

No data available.
